# Treatment with Statins in Elderly Patients

**DOI:** 10.3390/medicina55110721

**Published:** 2019-10-30

**Authors:** Ruxandra-Nicoleta Horodinschi, Ana Maria Alexandra Stanescu, Ovidiu Gabriel Bratu, Anca Pantea Stoian, Daniel George Radavoi, Camelia Cristina Diaconu

**Affiliations:** 1 University of Medicine and Pharmacy “Carol Davila”, 020021 Bucharest, Romania; ruxy691@yahoo.com (R.-N.H.); alexandrazotta@yahoo.com (A.M.A.S.); ovi78doc@yahoo.com (O.G.B.); ancastoian@yahoo.com (A.P.S.); radadaniel@yahoo.com (D.G.R.); 2 Clinical Emergency Hospital of Bucharest, 14461 Bucharest, Romania; 3 Academy of Romanian Scientists, Emergency University Central Military Hospital, 010825 Bucharest, Romania; 4 Clinical Hospital “Prof. Dr. Th. Burghele”, 050659 Bucharest, Romania

**Keywords:** elderly, atherosclerosis, statin, myocardial infarction, stroke

## Abstract

Elderly patients are a special category of patients, due to the physiological changes induced by age, the great number of comorbidities and drug treatment and last, but not least, to the cognitive dysfunction frequently encountered in this population. Cardiovascular disease is the most important cause of morbidity and mortality in elderly individuals worldwide. The rate of cardiovascular events increases after 65 years in men and after 75 years in women. Myocardial infarction and stroke are the leading disorders caused by atherosclerosis, that lead to death or functional incapacity. Elderly people have a greater risk to develop atherosclerotic cardiovascular disease. The incidence and prevalence of atherosclerosis increase with age and the number of cardiovascular events is higher in elderly patients. The most efficient treatment against atherosclerosis is the treatment with statins, that has been shown to decrease the risk both of stroke and coronary artery disease in all age groups. The advantages of the treatment become evident after at least one year of treatment. Primary prevention is the most important way of preventing cardiovascular disease in elderly individuals, by promoting a healthy lifestyle and reducing the risk factors. Secondary prevention after a stroke or myocardial infarction includes mandatory a statin, to diminish the risk of a recurrent cardiovascular event. The possible side effects of statin therapy are diabetes mellitus, myopathy, and rhabdomyolysis, hepatotoxicity. The side effects of the treatment are more likely to occur in elderly patients, due to their multiple associated comorbidities and drugs that may interact with statins. In elderly people, the benefits and disadvantages of the treatment with statins should be put in balance, especially in those receiving high doses of statins.

## 1. Introduction

Cardiovascular disease is the most important cause of morbidity and mortality in elderly individuals worldwide [1]. The rate of cardiovascular events increases after 65 years in men and after 75 years in women [1]. Myocardial infarction and stroke are the leading disorders caused by atherosclerosis, that lead to death or functional incapacity [2].

Elderly patients are a special category of patients, due to the physiological changes induced by age, the great number of comorbidities and drug treatment and last, but not least, to the cognitive dysfunction frequently encountered in this population. 

According to the World Health Organisation, elderly individuals are aged more than 60–65 years old [3]. Those above 75 years are considered old people and those above 90 years lifelong age [3]. Elderly people have a greater risk to develop atherosclerotic cardiovascular disease. The incidence and prevalence of atherosclerosis increase with age and the number of cardiovascular events is higher in elderly patients. Taking into account the great percentage of older people in the total population, it is very important to reduce the burden of atherosclerotic cardiovascular disease in elderly individuals. The most efficient treatment against atherosclerosis is the treatment with statins, that have shown to decrease the risk both of stroke and coronary artery disease in all age groups [4]. At the age of 65 years old, life expectancy is estimated to be more than 20 years in women and more than 17 years in men [5].

However, elderly individuals have a shorter life expectancy and more comorbidities than younger people, so statins may have fewer benefits in this population. This is why in elderly people, the benefits and disadvantages of the treatment with statins should be put in balance, especially in those receiving high doses of statins. 

The cardiovascular death is more frequent in women (55%) than in men (45%) in all age groups, but in people younger than 65 years, cardiovascular death is more frequent in men than in women [5]. The therapeutic options for older individuals have improved considerably, leading to an improvement in prognosis. Statins have conclusively proved their efficiency in reducing cardiovascular risk. Statins therapy in patients aged more than 65 years old decreases the risk of major cardiovascular events by 19%, which is similar to the reduction in patients younger than 65 years old, of 22% [1]. The advantages of the treatment become evident after at least one year of treatment ^1^. 

The Heart Protection Study included more than 20,000 people with cardiovascular disease or diabetes, who received simvastatin 40 mg daily versus placebo [6]. The risk of cardiovascular events was reduced by 18% in subjects aged 70 to 80 years treated with simvastatin and by 24% in those aged less than 65 years [6]. The authors observed a small difference in reducing the risk of cardiovascular events between elderly and middle-aged subjects, but the fact that elderly individuals have a higher risk of cardiovascular events at baseline, before starting statin therapy, should be taken into account. 

## 2. LDL (Low-Density Lipoprotein)—Cholesterol Target Values

Ideally, low-density lipoprotein (LDL)-cholesterol values in statin-treated patients should be less than 70 mg/dL [7]. In the PROSPER study, about half of the patients on pravastatin 40 mg daily had an LDL-cholesterol more than 100 mg/dL, which showed atherosclerosis progression and the lack of pre-existent atherosclerotic plaque stabilization [8]. The aim of the PROSPER study was to test the benefits of the treatment with pravastatin in patients with a history of vascular disease–cardiovascular disease, stroke (secondary prevention group), or with a high risk of developing vascular disease (primary prevention group) [8].

The drugs that decrease LDL-cholesterol more than 50% are rosuvastatin 20 to 40 mg, atorvastatin 40 to 80 mg, and simvastatin 20 to 80 mg [1]. 

The majority of the atherosclerotic cardiovascular events are nonfatal myocardial infarction or stroke. This is the reason why the proportion of patients with chronic atherosclerosis is increasing, meaning people who need statin therapy to prevent a second cardiovascular event. Real-life data show that in elderly subjects the maximum doses of statins are less likely to be prescribed than in younger patients [1]. A moderate dose of statin in elderly subjects may be preferred, to be well tolerated [1]. 

## 3. Primary Prevention

Primary prevention is perhaps the most important way of preventing cardiovascular disease in elderly individuals, by promoting a healthy lifestyle and reducing the risk factors, such as smoking, lack of physical exercise, a diet rich in fats, salt, obesity, alcohol consumption, early in life. A healthy lifestyle decreases significantly the risk of atherosclerosis. The treatment with statins reduces the risk both of myocardial infarction and stroke [9,10]. People who may benefit from preventive therapy should have a life expectancy of at least five years.

In the guidelines, patients are grouped in three categories of age: middle age (between 40 and 65 years old), elderly (between 66 and 75 years old), and very elderly (more than 75 years old). Primary prevention in the middle-age group consists of initiation of statin treatment in those at high risk for cardiovascular disease, as a class I recommendation [5].

Primary prevention in elderly individuals is also based on statin treatment in patients at high risk as a class I recommendation [5]. Therapy with statins reduces the relative risk of atherosclerotic cardiovascular disease in elderly patients similarly as in younger individuals [5]. Primary prevention reduces the risk of stroke and myocardial infarction but does not influence all-cause mortality and cardiovascular mortality [5]. According to JUPITER and HOPE-3 trials, rosuvastatin reduces significantly the risk of combined outcomes, consisting of nonfatal myocardial infarction, nonfatal stroke, and cardiovascular death by 49% in patients between 65 and 70 years old, and by 26% in those more than 70 years old [11,12]. 

In patients more than 75 years old, only the National Institute for Health and Care Excellence (NICE) guideline recommends initiating statin treatment as primary prevention [13]. On the other hand, the ACC/AHA and CCS guidelines provide that for people above 75 years old there are few pieces of evidence to sustain statin treatment [14,15]. 

Primary prevention is preferable to secondary prevention (after a cardiovascular event had already occurred), regarding the patient prognosis and the cost of the treatment [16]. The clinician should evaluate the advantages and the association of other side effects of the treatment with statins when initiating the primary prevention. The risk of side effects is influenced also by comorbidities and by the association of other drugs. 

In elderly people, it is more important to evaluate the risk-benefit balance, because they have more comorbidities and usually receive a complex treatment, with different classes of drugs, which may interact with statins, leading to a higher risk of potential side effects. On the other hand, elderly individuals have a shorter life expectancy, which may limit the potential advantages of statin therapy. This is another reason to evaluate rigorously the benefits and the risks and to prescribe statins with caution. 

## 4. Secondary Prevention

Secondary prevention after a stroke or myocardial infarction includes mandatory a statin, to diminish the risk of a recurrent cardiovascular event [17].

The Prospective Study of Pravastatin in the Elderly at Risk (PROSPER) included 5804 patients, aged between 70 and 82 years old, with a history of vascular disease or with a high risk to develop cardiovascular disease, due to hypertension, smoking, and diabetes mellitus. More than half were women [8]. The purpose of the study was to evaluate the advantages of prescribing pravastatin in elderly patients with, or at high risk of developing cardiovascular disease and stroke. Patients received pravastatin 40 mg daily or placebo. In individuals treated with pravastatin, it was observed that the risk of developing cardiovascular disease, including nonfatal myocardial infarction, cardiovascular death, fatal, and nonfatal stroke, has been reduced with 15% compared to placebo [7]. The level of LDL-cholesterol was reduced in 34% of the patients who received pravastatin [8]. 

The SAGE trial—Study Assessing Goals in the Elderly—compared the therapy with atorvastatin 80 mg daily with pravastatin 40 mg daily [18]. Patients treated with atorvastatin had lower all-cause mortality, but a non-significant decrease in major cardiovascular events. Another study—Heart Protection Study (HPS)—compared the treatment with simvastatin with placebo for five years [6]. It was noticed a reduction of 18% regarding coronary death and 25% regarding coronary events. The individuals were divided into three categories of ages—less than 65 years old, between 65 and 70 years old, and more than 70 years old—and there were no significant differences between the ages’ categories. The treatment with pravastatin had similar results [6]. 

Another cohort study included 23,000 patients with myocardial infarction treated with statins [19]. After three years of follow-up, in subjects aged 65 to 79 years, it was noticed a decrease of 11% in mortality rate, and in those aged more than 80 years any notable advantage on mortality rate was observed [19].

Stroke represents more than half of cardiovascular events in elderly women and almost half in elderly men [1]. Carotid atherosclerosis is one of the most important causes of stroke. The benefits of statins in patients with stroke were analyzed in several clinical trials. The SPARCL trial (Stroke Prevention by Aggressive Reduction of Cholesterol Levels) included 4731 patients, followed-up for five years [20]. Patients had a prior stroke or transient ischemic attack but did not have chronic heart disease. They received atorvastatin 80 mg daily. It was noticed a 64% higher risk of hemorrhagic stroke in patients who received atorvastatin than in the placebo group. However, the overall risk of recurrent stroke was diminished by 16%, due to the reduction by 21% of ischemic stroke. The mean level of LDL-cholesterol was 73 mg/dL in the atorvastatin group, while in the placebo group was 129 mg/dL. Patients were relatively young, with a mean age of 63 years. Patients had controlled blood pressure, with values between 138 and 139 mmHg for systolic blood pressure and 81 to 82 mmHg for diastolic blood pressure [20].

TNT (Treating to New Targets) trial included patients with chronic heart disease, with a mean age of 61 years and the blood pressure of about 131/78 mmHg [21]. Patients received atorvastatin 10 mg daily or 80 mg daily. There were no significant differences between the two groups [21]. 

Statins should be recommended as primary prevention in patients with asymptomatic carotid atherosclerosis and in those with a prior stroke as secondary prevention. The options of treatment for secondary prevention of ischemic stroke or transient ischemic attack caused by atherosclerosis include revascularization, especially for patients with symptomatic internal carotid artery atherosclerosis and medical therapy with statins, antihypertensive drugs, and antiplatelet agents to diminish the risk factors. 

Carotid endarterectomy is recommended in symptomatic patients with carotid stenosis. Statins are indicated in patients undergoing carotid endarterectomy, before and after the intervention. Symptomatic patients who receive statin treatment before the intervention may have a better outcome, a lower rate of in-hospital mortality and ischemic stroke [22]. On the other hand, in patients with asymptomatic carotid stenosis, statins are not connected with significantly different outcomes [23]. Another option for symptomatic patients with carotid stenosis is carotid artery stenting. These patients should also receive long-term statin therapy before and after revascularization.

## 5. Statin Therapy in Patients with Heart Failure

Apart from their effects in patients with cardiovascular ischemic disease, statins have anti-inflammatory effects, which may be beneficial in patients with heart failure with preserved ejection fraction (HFpEF) [24,25]. HFpEF develops especially in elderly individuals and 20% to 40% of elderly patients have isolated diastolic heart failure [24]. While statins seem to have no advantage in heart failure with reduced ejection fraction (HFrEF), in HFpEF statins may have benefits due to their anti-inflammatory properties. Several studies, such as CORONA and GISSI-HF, reported that statins have no advantages in HFrEF, with or without ischemic heart disease [24]. On the other hand, in patients with HFpEF, statins have benefits independent of the presence of ischemic heart disease [24]. In patients with heart failure with mid-range ejection fraction, statins have also some benefits regarding the reduction of cardiovascular events in those who associate ischemic heart disease. 

HFpEF is closely related to aging and comorbidities that induce a systemic and microvascular pro-inflammatory status, such as diabetes mellitus, hypertension, obesity, chronic obstructive pulmonary disease [25]. Thus, statins may be potentially beneficial in these patients. HFpEF is also a proinflammatory status apart from these comorbidities [26]. Statins may diminish the inflammation in HFpEF [24]. Statin treatment is related to reduced cardiovascular and all-cause mortality and hospitalization in patients with HFpEF [24]. 

## 6. Statin Therapy in Patients with Peripheral Arterial Disease

Lipid-lowering treatment with a moderate or high dose of statin is indicated in all patients with peripheral arterial disease (PAD). Patients with PAD are at a high risk of a major cardiovascular event, including heart attack, stroke, acute limb ischemia. Statin therapy reduces the risk of peripheral vascular events in patients with pre-existing PAD, but also in those at high risk, but who had not been diagnosed with PAD. For example, simvastatin 40 mg daily decreases the risk of myocardial infarction, stroke, or revascularization by about one-third and the risk of peripheral vascular events by about one-quarter [27]. In patients with diagnosed PAD, there was observed a significant decrease in vascular events, of about 22% [27]. There were also observed a reduction in the necessity of revascularization of 16% in the case of non-coronary revascularization and 30% lower rate of coronary revascularization [27]. The value of blood lipids before the treatment seems to have no importance in the efficacity of the treatment with statins [27]. In conclusion, statins should be considered in both patients with PAD or with a high risk to develop PAD. 

## 7. Statin Therapy in Patients with Chronic Kidney Disease

Usually, the management of dyslipidemia to decrease the cardiovascular risk is similar in patients with chronic kidney disease (CKD) as in patients without CKD. Patients with diagnosed atherosclerotic cardiovascular disease, including coronary, peripheral, or cerebrovascular disease should receive statin treatment with the maximally tolerated dose. Atorvastatin or fluvastatin are usually recommended because there is no need to adjust the dose according to the glomerular filtration rate [28]. In patients without diagnosed atherosclerotic cardiovascular disease, it is indicated a moderate dose of statin, such as atorvastatin 20 mg daily [28]. 

In CKD patients on dialysis, statin therapy should not be initiated. In hemodialysis patients, there were noticed no clinical improvement, although LDL-cholesterol values decrease [28]. In patients with end-stage renal disease, about 60% of cardiac deaths occur due to arrhythmias and sudden death, that is why lipid-lowering therapy is less important in these patients [28]. In patients already on statin therapy who need dialysis, the treatment can be continued [28,29].

## 8. Statin Therapy and Cognitive Function

Cerebrovascular disease can be located also in small cerebral arteries, defined as arteriosclerosis that leads to lacunar stroke and cognitive dysfunction, from mild cognitive impairment to vascular dementia. Vascular dementia is caused by cerebrovascular disease or impaired cerebral blood flow. Vascular dementia can be caused by any cerebrovascular or cardiovascular disease that affects the vascularisation of the brain, including large artery atherosclerotic disease, cerebral small vessel disease, cardio-embolism. The major risk factors for vascular dementia are the general risk factors for vascular disease: Advanced age, hypertension, elevated cholesterol levels, diabetes mellitus, smoking, coronary artery disease, atrial fibrillation, lower physical activity, obesity. Lipid-lowering treatment with statins to reach an LDL-cholesterol less than 70 mg/dL may be useful in preventing cerebrovascular disease, including dementia development [30]. 

## 9. Statins and Non-Alcoholic Fatty Liver Disease (NAFLD)

Non-alcoholic fatty liver disease (NAFLD) is characterized by hepatic steatosis in the absence of excessive alcohol intake, which may progress to cirrhosis. The management for patients with NAFLD includes: abstain from alcohol, vaccination for hepatitis A and B virus in those without serologic immunity, controlling cardiovascular risk factors, such as hypertension, hyperlipidemia, controlling blood glucose level in patients with diabetes. Statin use in patients with chronic liver disease is controversial because statins are metabolized in the liver, that’s why they can cause liver dysfunction. Liver disease is defined as an unexplained aminotransferase level more than three times the upper normal range on repeated tests. Statins are contraindicated in patients with acute liver disease, decompensated cirrhosis. When we initiate statin treatment in patients with chronic liver disease we recommend complete alcohol abstinence, we start with the minimum dose and we evaluate the impact on the level of aminotransferases in 4–12 weeks. Pravastatin and rosuvastatin are the preferred statins in these patients because they are not metabolized only by the liver [31]. In patients with progressive liver disease statins are discontinued. Concluding, statins should be administered with caution in patients with chronic liver disease and aminotransferases should be carefully monitored during the treatment.

## 10. Side Effects of the Statin Treatment

The main side effects of the therapy with statins are musculoskeletal disorders and diabetes, in all age groups (Table 1). 

Myopathy is characterized by unexplained weakness and muscle pain and creatine kinase level 10 times higher than the upper normal limit. Rhabdomyolysis is a more severe form of myopathy, with creatine kinase 40 times higher than the upper normal limit. Rhabdomyolysis usually requires hospitalization, because it can induce myoglobinuria and acute renal failure. The risk of rhabdomyolysis in patients receiving statins is reduced, at about 0.01% [32]. Muscular disorders are reversible with the interruption of statin treatment [32]. The risk is higher in the first year of treatment, after a dose increase. Usually, muscle pain or discomfort is not related to the treatment with statins, but patients perceive the symptoms as induced by statins because they are informed of statin’s side effects when they start the therapy. Rare cases of myopathy, with rhabdomyolysis, have been reported, and these cases are more frequent in the elderly than in younger people. Myopathy usually appears in patients who receive high doses of statins, which lead to higher plasma levels of active metabolites of the statins; it has been reported especially in those taking simvastatin 80 mg daily. For example, in Heart Protection Study the risk for myopathy was about 0.1% for simvastatin 40 mg daily compared with placebo [6]. Another trial—Study of the Effectiveness of Additional Reductions in Cholesterol and Homocysteine, which included 12,064 patients, revealed a risk of about 0.9% for simvastatin 80 mg daily and 0.02% for simvastatin 20 mg daily [33]. Still, even high doses of statins, such as atorvastatin 80 mg daily or simvastatin 20 to 80 mg daily, seem to have minimum side effects [32]. Side effects appear more frequently in older individuals and in patients with associated comorbidities and polypharmacy, such as hepatic failure, renal failure, pre-existing muscle disease, hypothyroidism, diabetes mellitus [32]. Moreover, female sex is a predisposing factor for myopathy [32]. 

Usually, myopathy appears a few months after the initiation of statin therapy or after a dose increase, manifesting with muscle weakness and pain [32]. The symptoms are bilateral and commonly are proximally distributed—shoulders, hips, upper chest, or lower back pain. The clinician should make the differential diagnosis with hypothyroidism, which is also associated with muscle weakness and creatine kinase’ increased values. 

Creatine kinase (CK) and transaminases should be measured in all patients presenting with muscle pain, as rapidly as possible, because myopathy can progress to rhabdomyolysis and acute renal failure. Statins should be stopped immediately if creatine kinase is more than 10 times the upper limit of normal, or more than five times in a critic patient, with very severe symptoms or with an associated disease that worsens his condition [32]. If CK is between three and four times the upper limit of normal and the symptoms are mild, the treatment may be continued, but the level of CK must be measured again in a few days. Patients should be well-hydrated. Typically, statin-induced myopathy is reversible when the treatment is interrupted, with the resolution of symptoms and a decrease of the creatine kinase level (Table 2). The time of recovery depends on the severity of the muscle injury. In case the symptoms do not disappear and creatine kinase does not decrease, the patient should be investigated for other pathologies, such as mitochondrial myopathies, polymyalgia rheumatica. A very rare disease is statin-induced autoimmune myopathy, which appears in two to three patients of 100,000 receiving statins [32]. This disease is variably reversible with statin treatment cessation. 

A cohort study, including 107,835 patients treated with statins, reported 11,124 patients who had interrupted the treatment, representing 10.3%, of whom 40% stopped the therapy because of muscular injury [34]. Of the patients who initially stopped the statin therapy, 6579 have restarted the treatment. After one year, 92% of them were still on statin treatment, which shows that the majority of patients tolerate the statins [34].

According to randomized trials, statins lead to a small increase of creatine kinase compared to placebo, for example, 8 U/L for rosuvastatin 20 mg daily, 20 U/L for atorvastatin 40 mg daily [32]. Concluding, myopathy is a rare side effect of statin therapy, related to the statin dose. Side effects appear especially at high doses of statins and in the presence of other drugs that interact with statin metabolism. Myopathy, including rhabdomyolysis, which is the most severe form, was observed in about 0.1% of patients receiving the maximal dose of statin [32]. When statin-induced myopathy is supposed, the therapy must be stopped immediately. After myopathy is excluded, statin therapy can be retaken with smaller doses or with another statin, to prevent atherosclerotic cardiovascular disease. 

Another possible side effect of the treatment with statins is diabetes mellitus, which is more frequent in elderly people, especially in old women [35,36]. Type 2 diabetes is a long-term disease, involving insulin resistance and loss of β-pancreatic cells, that takes place during many years. The incidence of the disease is higher in patients receiving statin treatment, who already have a pre-existent predisposition for diabetes, such as obese patients, with metabolic syndrome [32]. 

Several studies analyzed the possibility to develop diabetes mellitus in patients taking statins. The WOSCOPS (West of Scotland Prevention Study) was a randomized, placebo-controlled study, for primary prevention, which included men aged 45 to 64 years, without diabetes mellitus at baseline, and compared the development of diabetes in patients on pravastatin 40 mg daily versus those on placebo. The duration of the study was five years. There were included 2999 individuals who received pravastatin and 1.9% of them (57 subjects) developed diabetes, while 82 of 2975 of those on placebo developed diabetes, meaning 2.8% [37]. Statins are recommended in patients with diabetes and hypercholesterolemia, to prevent cardiovascular events. Statin therapy was associated with fewer hospital admissions for noncardiovascular complications of diabetes. This raises the possibility that although statin use may affect blood glucose levels, this does not necessarily lead to noncardiovascular diseases. We should note however that WOSCOPS was unusual, in that the incidence of type 2 diabetes mellitus was lower in patients who received statins.

The JUPITER prospective study included 17,802 subjects without diabetes mellitus at baseline and the outcome of the study was newly diagnosed diabetes in patients receiving rosuvastatin versus placebo [12]. The risk of developing diabetes was 0.6% higher in patients on rosuvastatin than on placebo, over a median time of 1.9 years [12]. Analyzing the results of the JUPITER trial, it was observed that statin therapy accelerates the onset of diabetes mellitus in subjects with metabolic syndrome or pre-diabetes [12]. 

Another study analyzed the possibility to develop diabetes mellitus in older patients with acute coronary syndrome, who should be prescribed intensive doses of statins [38]. Ko et al. observed only a small increase of the newly diagnosed diabetes rate in older patients who received intensive doses of statins, compared to those who received moderate doses, which is not statistically significant [38]. On the other hand, patients with an intensive dose of statins had a much lower risk for recurrent acute coronary syndrome than those treated with moderate doses of statins [38]. Taking into account that the cardiovascular risk significantly increases after statin therapy cessation, statins should not be stopped when diabetes develops [38]. This is why it is not established if diabetes is reversible with the interruption of the treatment because in the majority of the cases, statins are continued after the onset of diabetes. 

Regarding individuals who already have diabetes when they start statin therapy, hemoglobin A1c level increases with 0.3% at four months, respectively 0.1% at four years of treatment, according to AFORRD and CARDS trials [39,40]. The AFORRD study included 800 patients with diabetes mellitus, who received atorvastatin 20 mg daily versus placebo [39]. The CARDS study compared the effect of atorvastatin 10 mg daily versus placebo in 2838 patients [40]. 

In conclusion, statins induce only a small increase in the risk of developing diabetes mellitus, especially in patients who already have multiple risk factors for diabetes. The risk of statin-induced diabetes in major clinical trials was about 0.2%/year [41]. The increase of the hemoglobin A1c in patients with diabetes is also small after initiation of statin treatment. Considering the important effect in diminishing cardiovascular risk in all patients, including those with diabetes mellitus, it is not recommended to stop the statin therapy in patients with pre-existing or newly diagnosed diabetes. Thus, diabetic individuals should receive statins to reduce cardiovascular risk [41].

Statins may also have liver side effects, especially in subjects who already have liver diseases because of alcohol use or fatty liver disease. The risk of serious liver toxicity is about 0.001% [32]. The TNT (Treat to New Targets) study included 10,001 individuals and compared hepatotoxicity of atorvastatin 80 versus 10 mg per day, for almost five years [21]. From patients receiving atorvastatin 80 mg daily, there were 60 cases of increased aminotransferases more than three times the upper limit of normal, while from patients receiving atorvastatin 10 mg per day there were only nine cases of increased aminotransferases [21]. This means that statin hepatotoxicity is dose-related. The EXCEL study, which included more than 8000 patients who received lovastatin or placebo, monitored for 48 weeks, evaluated the risk of developing hepatotoxicity, defined as an increase of alanine aminotransferase or aspartate aminotransferase greater than three times the upper limit of normal [42]. For lovastatin 20 mg daily the risk was the same with placebo—0.1%, but for higher doses of lovastatin 40 or 80 mg daily, the elevation of aminotransferases was higher in patients treated with lovastatin, 0.9%, respectively 1.5% [42]. The JUPITER trial also monitored hepatic injury [12]. This trial included approximately 18,000 subjects who were randomized to rosuvastatin 20 mg versus placebo [12]. The rate of hepatic injury was 2.4% in subjects on rosuvastatin and 2.1% in those on placebo. As we notice, the difference between rosuvastatin or placebo hepatic injury risk is insignificant. In the Heart Protection Study, which compared simvastatin 40 mg with placebo in more than 20,000 individuals for five years, there were only six cases of hepatitis in the group designated to simvastatin and nine cases in those on placebo [6]. Therefore, according to major randomized clinical trials, the risk of hepatic injury in patients treated with statins is very modest. Severe liver disease induced by statins was reported only in case reports. Statins may cause small, asymptomatic aminotransferases elevations. These elevations may be greater than three times the upper limit of normal in about 1% patients, especially in patients with pre-existing liver disorders, and this does not mean certainly hepatic injury [32]. 

Intracerebral haemorrhage is a controversial possible side effect of statin therapy. According to the majority of the clinical studies, there is no association between statin use and intracerebral haemorrhage [43]. Moreover, there is no evidence that a greater degree of lowering the cholesterol level would be related to a greater risk of intracerebral haemorrhage. According to the SPARCL trial, even patients with a minimum 50% decrease of LDL-cholesterol level were at no higher risk of intracerebral haemorrhage than patients whose LDL-cholesterol increased or had no change during the trial [44].

## 11. Conclusions

Taking into account the continuous increase of life expectancy, preventing atherosclerotic cardiovascular disease and its consequences on the quality of life has become a very important objective in elderly patients. Statin treatment is effective in both primary and secondary prevention of cardiovascular events [45]. Statins should be prescribed with caution in elderly individuals, who may present more frequent side effects of the treatment than younger people, because of the multiple associated comorbidities and drug interactions. In conclusion, despite the possible side effects of the therapy, elderly patients should receive statins to avoid a cardiovascular event, such as myocardial infarction or stroke. Prevention of a first or recurrent cardiovascular event is the most important goal of statin therapy, which may considerably improve morbidity and mortality in older people [46]. 

## Figures and Tables

**Table 1:** Statins side effects.

Musculoskeletal Disorders	Diabetes	Liver Side Effects	Intracerebral Hemorrhage
Myopathy	More frequent in elderly patients, especially women	Very modest Dose-related	Controversial possible side effect
Rhabdomyolysis	It appears especially in patients with multiple risk factors for diabetes	Usually small, asymptomatic aminotransferases elevations	The majority of studies found no association between statin treatment and intracerebral hemorrhage

**Table 2:** The management of cases with myopathy as statin treatment side effect.

CK	>10 x N	STOP the statin
CK	3–4 x N mild symptoms	Continue the treatment Check CK again in a few days Good hydration of the patient

CK: Creatinkinase.
